# Perceived barriers and facilitators for physical activity in youth and young adults living with hemophilia. A focus group study

**DOI:** 10.3389/fspor.2026.1879362

**Published:** 2026-08-11

**Authors:** Tabea Nauschütz, Ulrich Theobald, Christoph Königs, Jonathan Knapp, Martin Giese

**Affiliations:** 1 Department of Educational Science and Sports Science, Marburg University, Marburg, Germany; 2 Faculty of Special Education, Ludwigsburg University of Education, Ludwigsburg, Germany; 3 Department of Paediatrics and Adolescent Medicine, Goethe University, Frankfurt am Main, Germany

**Keywords:** ableism, focus groups, hemophilia, inclusion, participation, physical activity

## Abstract

**Introduction:**

Hemophilia is a rare hereditary bleeding disorder characterized by a deficiency of clotting factor, resulting in an increased bleeding tendency. Historically, people living with hemophilia (plwh) were advised to avoid physical activity (PA) due to injury risk. Today, improved treatment options enable plwh more self-determined lifestyles. Moreover, medical perspectives on PA have shifted underlining the benefits of regular engagement in PA for physical and mental health. Nevertheless, youth and young adults living with hemophilia (yyalwh) show comparatively low exercise capacity and lack in sport-specific motor performance. Research on experiences and perspectives of plwh regarding participation in PA remains limited. Therefore, this qualitative focus group study aims to explore perceived barriers and facilitators to participation in PA among yyalwh.

**Methods:**

Seven focus groups were conducted with 28 participants aged 14–30 years with different types and severities of hemophilia. Data were analyzed using a structuring qualitative content analysis approach. Factors perceived as supporting participation were categorized as facilitators, whereas factors perceived as hindering participation were categorized as barriers.

**Results:**

Five main categories related to participation were identified across multiple levels: individual, social, environmental, policy, and societal. Barriers and facilitators were found across all levels and interacted with each other. The social level emerged as particularly influential. Support from family members, doctors, and teachers was described as a key facilitator, while explicit restrictions negatively affected engagement. The societal level also proved to be highly relevant, since it interacts with the other levels and reflects ableist narratives that yyalwh face in everyday life.

**Discussion:**

Overall, participation in PA and sports among yyalwh is shaped by complex interrelations of barriers and facilitators across these levels. The findings may contribute to raising awareness of these dynamics and highlight the importance of involving yyalwh early in self-determined decision-making processes.

## 1 Introduction

In 2006, the Convention on the Rights of Persons with Disabilities (UN-CRPD) was adopted by the United Nations General Assembly and entered into force in 2008. The UN-CRPD aims to strengthen the rights of persons with disabilities (pwd) and reduce social exclusion (1). Despite this international commitment, people with disabilities and/or chronic illnesses continue to face numerous barriers across different life domains, including employment (2), education (3) and leisure (4). One domain in which inequalities become particularly evident is physical activity (PA), defined as ‘any bodily movement produced by skeletal muscles that requires energy expenditure’ (5), and sports understood as rule-governed activities carried out in context of leisure or competition, often supported by organizational structures (5). Pwd participate in PA and sports less frequently than those without disabilities (6, 7). A report published 2021 by the United Nations further highlights that pwd encounter numerous barriers, ranging from structural-organizational barriers, such as inaccessibly physical environments or a lack of accessible information, to social barriers, including overprotectiveness and prejudices (8). Participation in sports is recognized as a right under Article 30 UN-CRPD, and in conjunction with Article 4, signatory countries are obliged to actively enable such participation. Beyond its relevance for physical and mental health, being physical active enables meaningful and formative experiences (9).

Against this background, people living with hemophilia (plwh) also experience barriers concerning sports participation (10–12). This pattern is intertwined with medical treatment, which is therefore briefly outlined in the following section.

Hemophilia is a rare hereditary bleeding disorder, characterized by a deficiency of factor VIII (hemophilia type A) or factor IX (hemophilia type B). Depending on the extent of the factor deficiency, the degree of severity ranges from mild to moderate to severe (13). While people with severe hemophilia frequently experience spontaneous bleeding, particularly in joints and muscles, and prolonged bleeding after minor and major trauma as well as surgery is a regular case, spontaneous bleeding in people with moderate hemophilia occurs only occasionally (14). In people with mild hemophilia, bleeding relates to trauma and surgery. Bleeding can lead to irreversible damage (hemophilic arthropathy), accompanied by swelling and pain, especially in the joints (15). Recurrent joint bleeding (hemarthrosis), particularly in knees, elbows and ankles, is a frequent clinical manifestation in people with severe hemophilia, even though recent data have revealed that also patients with moderate and mild severity (although less frequently) are affected (16). In addition to (irreversible) physical restrictions, joint bleedings cause pain, significantly affecting the individual quality of life of plwh (17).

For decades, sports participation had been discouraged due to injury risk accompanied by a bleed and less efficacies therapies (18, 19). In the last years, the medical perspective on PA and sports among plwh has shifted towards promoting participation (20). This change is closely linked to advances in treatment (21). Current lifelong treatment focusses on prophylactic, preventive therapy with additional treatment in case of bleeding events or surgery (22). Whereas plwh once depended on frequent hospital-based care, those born today in high-income countries typically grow up practicing independent home-based self-treatment, often supported by mobile technology (23). At the same time, the importance of regular PA for maintaining physical health is now widely recognized, inter alia to prevent further chronic conditions and to promote mental well-being (24). Consistent with this, the World Federation of Hemophilia (WFH) explicitly encourages participation in PA and sports while still providing recommendations on suitable and unsuitable activities and appropriate protective practices (25). Despite these developments, empirical research points to ongoing challenges: an increased prevalence of overweight and obesity in young people living with severe hemophilia has been reported (26). In addition, a review by Cruz-Montecinos et al. (27) has shown that the physical performance of yyalwh is lower than that of their peers without hemophilia, while Seuser et al. (28) identified deficits in the sport-specific motor performance of yyalwh.

Beyond medical considerations, participation in PA and sports for plwh is also shaped by social dynamics (29) as well as an increased fear of movement and kinesiophobia (12). Hemophilia is not visible from the outside, which means that individuals living with hemophilia can move through society largely unrecognized (30). However, regular medical consultations remain essential and frequently involve risk assessments framed through a deficit-oriented lens. This framing shapes parental, educational, and medical decisions and often restrict children and adolescents opportunities to participate in sports (30, 31). Repeated joint bleeds and inflammation can definitely cause long-term damage (16), but the social consequences of risk-framing may also be profound: many plwh report experiences of exclusion, overprotection, and restricted autonomy in sports throughout their lives (10, 30). Matlary et al. (32) outline in their review that influences on PA for plwh are manifold. The authors identify possible facilitating and hindering factors at individual, social, environmental and policy level and explicitly address clinicians, highlighting the need for individualized advice. This clinical focus must be supplemented by an equally relevant perspective, namely that of the plwh themselves.

Furthermore, existing research on PA and sports among plwh is largely designed quantitatively, addressing whether PA occurs rather than how it is experienced (33, 34). As a result, the subjective voices of plwh, especially their socially negotiated experiences of inclusion and exclusion in sports, remain almost entirely absent from literature. Yet, these subjective voices are crucial for understanding the reasons for sports abstinence from an intersubjective perspective. Similar patterns have been found in research on people with visual impairment (35, 36); therefore, the following sections refer to findings from this field. To the best of our knowledge, only one qualitative study has explored reasons for (non-)participation in sports activities among plwh. This study focuses exclusively on adults (11), even though many exclusionary experiences originate in childhood and have a crucial influence on the further sports biography (37).

Our study addresses the aforementioned gap and aims to explore socially shared experiences of barriers and facilitators of PA and sports participation among yyalwh. This results in the following research question: How do yyalwh perceive barriers and facilitators of participation in PA and sports? Using data from qualitative focus group, the study examines how yyalwh negotiate PA within the tension between medical risk narratives, autonomy and social interactions, and offers insights into their articulated desires for more equitable participation. To begin with, the study's methodology for qualitative data collection and analysis will be explained, followed by a presentation and discussion of the results. Finally, the study's limitations and a conclusion are provided.

## 2 Methodology

### 2.1 Research design

The present focus group study adopts a qualitative design grounded in an interpretivist epistemology, meaning that the subjective experiences described by the participants are understood by the researchers as something meaningful and that the researchers play a critical role in the co-construction of knowledge (38). Rather than aiming to establish generalized truths about barriers and facilitators, our goal is to reconstruct and in-depth understand the effects of subjectively perceived and socially shared experiences of barriers and facilitators in PA and sports participation of yyalwh. Focus groups were chosen for their flexibility and their capacity to surface collectively negotiated meanings through discussions (39).

The study received approval from the ethics committee of the medical faculty of [Goethe University]. Prior to participation, all participants (including their parents in the case of minors) were informed about the research interest of the study, gave their written consent and were assured that their personal data and confidentiality would be preserved. The study is presented in accordance with the consolidated criteria for reporting qualitative research (COREQ) (40).

### 2.2 Participants & research setting

The study employed a specific recruitment system, resulting in a selected sample (41). Specifically, participant recruitment relied on the temporal and organizational structures of the [Just-Move-It] project, a collaborative project of different stakeholders organized by the [Goethe University]. Consequently, the sample was limited to yyalwh who participated in the project. The project spanned a total of six months and was open to yyalwh aged 14–25. One participant older than the target age range was exceptionally admitted due to a strong interest in participating in the project. The project included two multi-day sports weekends (11/2024; 05/2025), which served as the project's start and end points.

These weekends, during which the qualitative focus groups took place, offered professionally supervised, participant-driven sports activities. Additionally, they included educational teaching formats addressing topics related to sports, hemophilia and nutrition, and created opportunities for peer interaction as well as shared physical experiences. The project team consisted of people with different professional backgrounds, specifically doctors, physiotherapists, and sports and educational scientists. During the sports weekends, the project team aimed to involve participants' preferences in the program to acknowledge them as experts in their own lives.

Between weekends, participants pursued their own physical activities and exchanged via shared apps, fostering continuity and community. The project was advertised through patient organizations and hemophilia treatment centers, resulting in a geographically distributed sample across Germany. Focus group recruitment took place on-site on a voluntary basis. Inclusion criteria were: (1) diagnosis of hemophilia, (2) minimum age 14 years, (3) sufficient language proficiency, and (4) consent to participate. The final sample comprised 28 male yyalwh aged 14–30. There was no disruption or drop-out from the sample.

The composition of the focus groups was largely determined by the participants themselves, which resulted in groups with varying age constellations. Participants were allowed to choose their discussion partners in order to create a comfortable atmosphere and encourage open conversation within the focus groups (39). The classification of participants as youth and young adults follows the categorization proposed by Potì et al. (15). In one case, the age distribution included an outlier, as one participant was 30 years old. However, this participant was retained in the sample despite being an age outlier, as he explicitly expressed a strong interest in participating in the focus group and contributing to the discussion (Table 1).

**Table 1:** Characteristics of participants.

Gender	Male (*n* = 28)
Age (years)	14–30 years (mean: 19,1)
Bleeding disorder	
Severe hemophilia A	*n* = 18 (64,29%)
Severe hemophilia B	*n* = 3 (10,71%)
Moderate hemophilia A	*n* = 6 (21,43%)
Moderate hemophilia B	*n* = 1 (3,57%)
Plwh receiving prophylactic treatment	*n* = 28 (100%)

### 2.3 Data collection

A total of seven focus group discussions were conducted in German with four to six participants per focus group. All audio recordings were digitally saved. Focus groups (FG) took place during both sports weekends in seminar rooms at the venues.

An interview guide, which was developed and discussed within the research team and pilot-tested for linguistic clarity and relevance of the topics by a young adult living with hemophilia, structured the moderation (42). It included open-ended questions on perceived barriers and facilitators within general physical activities and physical education (PE) classes in school, as well as desires regarding (future) sports participation. Following the flexible nature of focus groups, the sequence and depth of topics varied depending on group dynamics. Discussions lasted between 36 and 80 min.

[T.N.] moderated all seven groups; [U.T.] co-moderated the first three. Both researchers have a university degree in sports and education science and have worked in practice and academia. They were involved in planning and implementing the sports weekends and interacted with participants outside the formal research setting. Participants were likewise familiar with each other, some had longstanding connections, others met during the project. Thus, the relationship between the participants and the researchers can be described as friendly and trustful.

### 2.4 Data analysis

All recordings were transcribed verbatim, translated in English and pseudonyms were assigned to protect participants’ personal data and identities. Data were analyzed using the structuring qualitative content analysis approach that combined deductive and inductive coding (43). To become familiar with the material, the transcripts were scrutinized repeatedly, and case memos were written documenting emerging impressions. Subsequently, two authors [(T.N. & J.K.)] coded the transcripts line by line to identify relevant themes and to organize them into main and subcategories.

**Figure 1:**
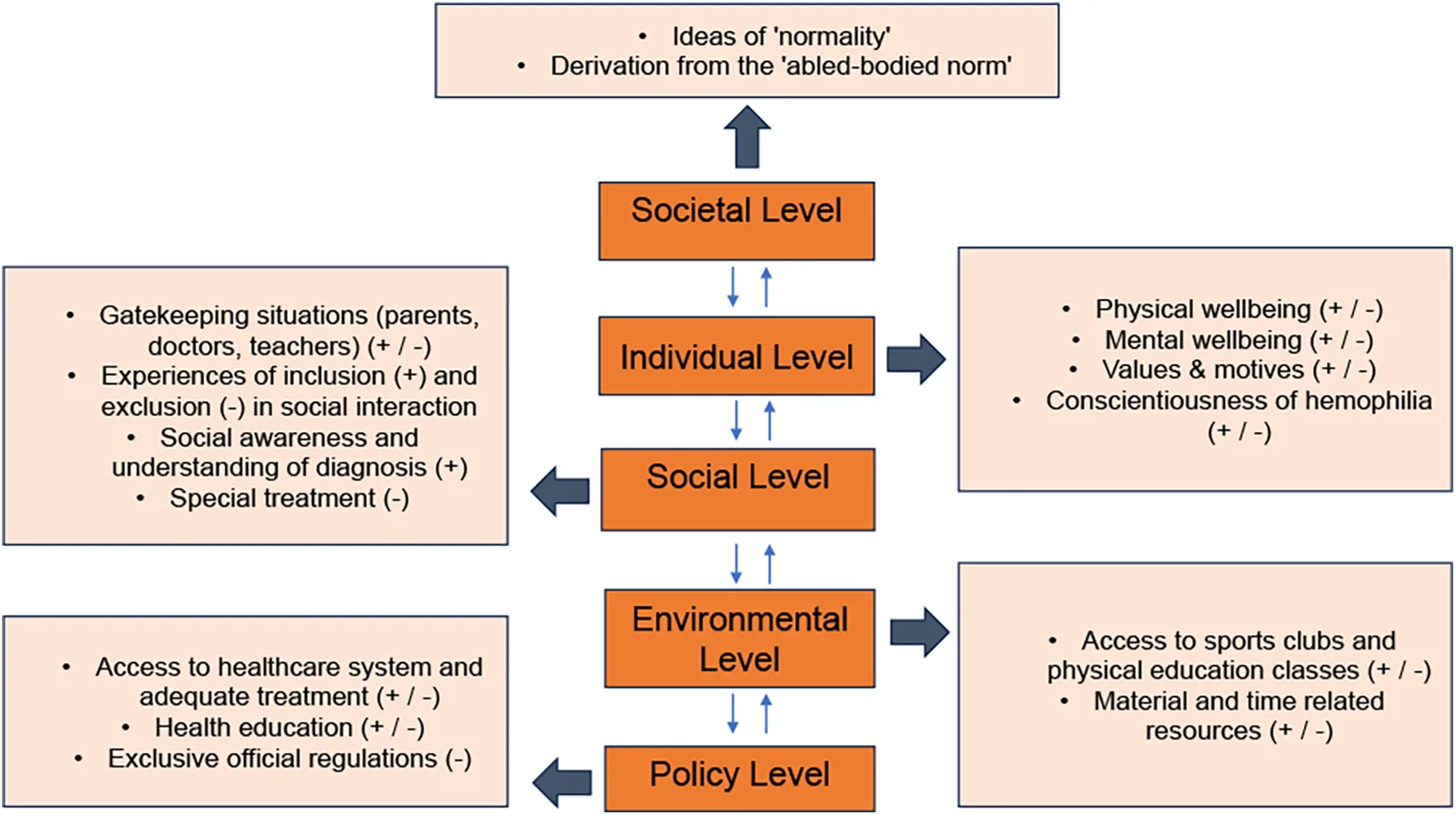
Code system adapted from Matlary et al. (32) [Promoting physical activity for people with hemophilia in the age of new treatments, hemophilia published by John Wiley & Sons Ltd., DOI: 10.1111/hae.14641]. Licensed under CC BY-NC.

Deductive coding was conducted theory-driven: Matlary et al. (32) illustrate four levels (individual, social, environmental, policy) of physical activity influences for plwh, which served as predefined main categories and guided the initial coding structure. During the circular coding process, another main category – the societal level – emerged. This category refers to previous research on physical activity and disability (36). Inductive coding was conducted data-driven: Within these overarching main categories, subcategories were developed directly from the focus group data to capture recurring themes grounded in participants’ voices. Within the subcategories, barriers and facilitators were identified and assigned to the corresponding levels. Factors perceived as supporting participation were categorized as facilitators, whereas factors perceived as hindering participation were categorized as barriers. While many of the identified subcategories can also be found in frameworks used by Matlary et al. (32) and Ruin et al. (36), only those that were explicitly evident in our empirical material and emerged as consistent patterns across different participants (i.e., mentioned repeatedly by multiple individuals) were included in the final coding system. Figure 1 shows the code system based on Matlary et al. (32), with the addition of the societal level. The arrows between the levels should be viewed as all components being in dynamic relationship with each other.

When defining categories, it is essential to continuously reflect on one's own prior knowledge (44). Thus, the code system was developed in close collaboration between all researchers. The entire process was supported by MaxQDA Analytics Pro (Version 24.6.0; VERBI GmbH Berlin), which facilitated data management (45).

### 2.5 Trustworthiness

To ensure the trustworthiness of the study, strategies were implemented throughout different stages of the research process (46). Within the constructivist paradigm, which considers researchers as co-constructors of knowledge, it is particularly important to reflect on the research team's prior knowledge and positionality (47). In addition to the methodological considerations outlined above, it should be noted that two authors (T.N. & C.K.) involved in the study had previous professional and personal experience with plwh. The remaining authors had no prior professional or personal experience with hemophilia, resulting in varying levels of hemophilia specific knowledge within the research team. Furthermore, all members of the research team identify as “able-bodied”. Besides other aspects, these dimensions can shape the researchers’ embodied experiences, knowledge, assumptions, values and language, and therefore require ongoing reflection throughout the research process, particularly during data analysis (48). Beyond these reflections on the research team's positioning, strategies were employed to further strengthen the trustworthiness of the study (46). Therefore, the methodological procedure was described in the Methodology section and supported by tables and figures. Credibility was enhanced through a description of the sampling process, participant characteristics, and the chosen data collection method. Dependability was addressed by describing methodological decisions and by specifying the authors involved in the coding. To enhance transparency and facilitate understanding of the findings, insightful quotes from the participants were used to illustrate the meanings of the categories. Transferability was considered by critically discussing the limitations arising from the selective sample. Finally, potential sources of bias and their implications for the interpretation of the findings are discussed in the Limitations.

## 3 Results

The findings provide insight into subjectively perceived and socially shared barriers and facilitators influencing and shaping sports participation among yyalwh. Although all focus groups followed the same guiding questions, the emphasis and depth of specific topics varied across groups. During the focus groups, participants frequently asked each other questions, which indicates a strong interest in shared experiences and opinions of peers living with hemophilia.

For the purposes of this paper, we primarily present recurring patterns from the data organized by the main categories. The inductive categories and anchor examples can be found in Table 2 for a more detailed picture. Results remain descriptive and do not aim to exhaust every detail of the dataset, which would neither be feasible nor methodologically appropriate.

**Table 2:** Code system with anchor examples. Subcategories can act as barriers (-) or as facilitating factors (+).

Main Category	Subcategory	Anchor example
Individual Level	Physical wellbeing	(+) William: I must say, I consider myself very lucky to have had very little to almost no joint bleeding so far. […] So I don't have any pain in my joints at all. (FG 04) (-) Laurence: When I play jumping or racing games, I usually end up experiencing pain. Even when I go to sleep, I often cannot fall asleep because of severe ankle pain. (FG 02)
	Mental wellbeing	(+) Michael: I would also say that I feel healthy. Because I can actually do what I want. I think that's part of it. When you do sports, you can do the sport you want. (FG 06) (-) Jasper: So I'll say it like it is: I'm a little afraid of the future, because if I get injured and can't do any sports at all, then I can already feel my head starting to go a little crazy. (FG 05)
	Values & motives	(+) Clement: For me, sport is definitely part of my life, and when I don't do any sport, I immediately notice that it has an impact on other parts of my life. Because it's also a kind of stress relief and it's just fun. That's why I couldn't imagine a life without sport now. (FG 01) (-) Oscar: That fits quite well, because for me it's exactly the opposite. For me, sport isn't a big part of my life. […] So, compared to others, I don't necessarily need a lot of compensation. (FG 04)
	Conscientiousness of hemophilia	(+) Jasper: But when you're in a training session or a game, I just forget everything. I play as if I don't have hemophilia and I go into tackles […] Yes, I went for the ball as if I didn't have anything to worry about. (FG 05) (-) Brandon: But I didn't enjoy it [skiing] either, because in the back of my mind I kept thinking, ‘Yes, if you twist your ankle or fall somehow, something bad could happen.’ And then it's not such a nice feeling all the time. (FG 02)
Social level	Gatekeeping situations (parents, doctors, teachers) (+ / -)	(+) Leo: For me, it was also my parents. They argued strongly for swimming and the gym, supported and helped me a lot. (FG 06) (-) Ben: So my mother doesn't want me to play soccer because she's worried about me. (FG 02)
	Experiences of inclusion (+) and exclusion (-) in social interaction	(+) Nick: I participated in everything. I was never excluded. I was always chosen first or second. That was the case in all sports. PE class was amazing. It was awesome. (FG 04) (-) Matthew: For example, when the class is playing soccer. You just feel excluded when you have to sit on the bench and wait until it's over. (FG 02)
	Social awareness and understanding of diagnosis (+)	(+) John: So at school, I just join in as normal, except if something hurts, then I go to my teacher and say that I can't take part. And they understand that. And then I just stay outside. (FG 05)
	Special treatment (-)	(-) Marcus: My mother once said, ‘Here, my boy, he has this and that.’ I had never had any problems before, but suddenly I was treated completely differently. Here at gymnastics. So, this typical jumping over a vaulting buck or something like that. ‘Here, you'd rather not do that, you'd better watch.’ (FG 07)
Environmental level	Access to sports clubs and physical education classes (+ / -)	(+) Theo: […] because I didn't really know how to train in a structured way, like making a plan and then doing it several times a week, but I ended up going to training five times a week and felt totally fulfilled. (FG 07) (-) Finnley: Then it just didn't go any further because there were no competitive events in the swimming club. (FG 01)
	Material and time related resources (+ / -)	(+) Laurence: I now have bandages on my foot, which means I can exercise better, have less pain, and have a more stable gait. That's exactly what's very helpful. (FG 02) (-) Oscar: And I mean, when I was at university, it was still possible to do sports in general, in terms of the amount of time. You just have a little more time, but since I've been working full-time, you can more or less forget it. (FG 04)
Policy Level	Exclusive official regulations	(-) Leo: In [country of birth], I was not allowed to go to school. I was excluded. (FG 06)
	Access to healthcare system and adequate treatment (+ / -)	(+) Steve: But in terms of what we actually still need or what is actually important, generally speaking, the availability of medication. Fortunately, this is not a huge issue in Germany at the moment. […] But we are talking about incredible amounts of money that somehow end up in our bodies. […] This should definitely continue to be the case. (FG 01) (-) James: For example, I have a friend from [country of birth] who played soccer without taking any prophylactic medication. Now he's 22 and can't walk straight anymore. So in my case: it was fine in Germany, but difficult in [country of birth]. […] It's reasonable that I wasn't allowed to do anything there. (FG 06)
	Health education (+ / -)	(+) Richard: And in terms of sports, we were given so much support from the treatment center anyway. […] There were constant opportunities presented to us, simply pointing out, ‘Hey, you can do this. […] Hey, this and that is possible.’ (FG 03) (-) Nick: And that's where you see again that in Germany, it absolutely depends on whichever center you find yourself at. You think it should be/ it is standard knowledge, but then you might be unlucky and end up at center X, where this knowledge, which we thankfully have, is not being taught. (FG 04)
Societal level	Ideas of normality	Steve: That normal people or non-hemophilia people simply learn to accept that. Yes, to accept it sounds a bit harsh, but you know, there isn't just the normal way, the healthy way, where you can do whatever you want. There are also other people, and they are normal too, but just differently normal, if you understand what I mean. (FG 01)
	Derivation from ‘normality’	Ben: And since I know myself that when I inject (name of medication), I am like a normal person for that period of time, I can actually do that [play soccer].’ (FG 02)

### 3.1 Individual level

This category referred to individual factors focusing only the level of the participants that influenced their PA and sports participation either positively or negatively.

At this level, descriptions of individual *physical* and *mental well-being* stood out. Physical and psychological components often interacted and influenced each other in their impact. This dynamic interaction became particularly evident in discussions about injuries. Participants reported different ways of dealing with injuries. For Scott, an injury ‘*actually has almost positive connotations*' (FG 03) because it provided an opportunity for reflection: ’*So when I have an injury, I realize, I haven't been taking enough care of myself lately. [..] And then the next week or two are blocked and it's all about me.’* (ibid.). Jasper expressed the opposite: ‘*I hate injuries because they always take so long to heal completely*.’ (FG 05). Later in the conversation, Jasper explained that he is afraid of injuries because they require a break from sports, which in turn lead to mental discomfort.

Furthermore, personal *values and motives* influenced participation in sports in both supportive and hindering manners. The sample was characterized by an affinity for sports, which was why positive attitudes toward sports and supportive motives predominated. Among other things, participants mentioned engaging in sports to cope with stress from school or work, pursue personal goals such as improving physical performance, and experiencing flow moments and enjoyment in sports participation.

Finally, the *conscientiousness for hemophilia* also played an important role at the individual level. Several participants reported that they occasionally completely forgot or rather ignored their hemophilia when participating in sports and consciously accepted potential consequences. On the other hand, some participants described behaving cautiously based on their experiences and knowledge and considering their actions carefully.

In summary, the findings suggest that at the individual level – depending on the situations described by the participants – emotions related to sports and hemophilia are primarily negotiated.

### 3.2 Social level

This category captured the role of social interactions and relationships in influencing (non-) participation in sports.

Participants described numerous *gatekeeping situations* involving actors in their immediate social environment, such as doctors, parents, other relatives, and teachers. It became evident that decisions made by parents and doctors significantly influenced participation in organized sports (club sports, school sports, varsity) as well as leisure-time physical activities. Explicitly supportive and liberating statements and behaviors from parents and doctors were highlighted as particularly positive. In contrast, purely prohibitive statements were described as negative. Participants pointed out that there were considerable differences in how actors in their immediate social environments behaved and that their own (later) management of hemophilia was significantly shaped by these social actors.

In addition, participants reported *experiences of inclusion and exclusion*. In the data, these situations were primarily described in relation to physical education (PE) at school. Several participants stated that they enjoyed participating in PE, felt part of the group, and were able to gain a wide range of physical experiences. On the other hand, moments of exclusion were described as having an impact not only at the time itself, but also beyond. Theo, who had been exempt from PE since the age of twelve, reported: ‘*And I just sat and watched [in PE classes] and then I noticed that a certain distance [from my classmates] had simply developed.*’ (Theo, FG 07).

*Special treatment*, especially from teachers who were perceived as overcautious, was also described as a negative factor influencing participation in sports. In line with this, participants perceived unrestrained curiosity and the urge to describe hemophilia on behalf of external and sometimes even unfamiliar actors as annoying and disruptive. In contrast, *awareness and understanding* from familiar actors, such as teachers, towards participants was generally described as supportive and beneficial.

Overall, it can be stated that numerous multidimensional and complex social situations were brought up by the participants themselves.

### 3.3 Environmental level

This category referred to material and structural factors that influenced opportunities for sports participation. Involvement in organized club training and competition was described as beneficial for regular participation in sports. Theo described how he appreciated the regular training schedule, and how it encouraged him to exercise regularly: ‘*[…] because I didn't really know how to train in a structured way, like making a plan and then doing it several times a week, but I ended up going to training five times a week and felt totally fulfilled*.’ (FG 07). Conversely, lack of sports programs for younger age groups, or a lack of time due to university studies or work represented barriers. In addition, participants in one focus group criticized the fact that access to PE classes may be limited by curricula that include sports not medically recommended for plwh.

When it came to practicing sports, supportive equipment such as bandages was highlighted as a facilitating factor that enabled participants to engage in physical activity more safely. In addition, some individuals were regularly involved in sports due to professional or educational contexts, such as studying sports science or working as a volunteer coach.

In total, the environmental level was less prominently represented in the data than the previous two levels and was often discussed in connection with the policy level, indicating a close link between these two.

### 3.4 Policy level

This category referred to factors related to *official regulations* issued by institutions as well as aspects of the health care system that influenced participation in sports.

For example, two participants who now live in Germany reported that they were excluded from PE class in their country of birth due to their hemophilia, and in one case even from school altogether. Several participants described *access to the healthcare system and adequate treatment* as a fundamental prerequisite for everyday life and, beyond that, for sports-related life. In this context, participants reflected critically on global inequality in the distribution of medical care. Steve shared a personal experience: ‘*We once had a [South American] exchange student staying with us, also a hemophiliac, and he told us that they somehow get medication once a month, and for them, it's basically like they save it up. That means he doesn't do any sports, and then they save up for two or three weeks so that if there is a bleeding, he can inject everything at once, and that just doesn't make any sense.’* (FG01). Steve further emphasized the importance of ensuring a continuous supply of medication for plwh, a view that was shared by other participants in the focus group. Furthermore, the participants viewed the continuous development of medications and treatment therapies positively, as these developments could shorten recovery times after injuries and enable a faster return to sporting activities.

Participants also considered *health education* programs to be particularly important. Opinions were divided regarding written brochures and information material addressing the topic of ’sports and hemophilia.’ Some participants used these materials as guidance, while others criticized what they perceived as rigid classification of certain sports as either suitable or unsuitable. Sports-related programs offered by medical care centers and patient organizations, as well as other projects such as [Just-Move-It], were also cited as helpful. Participants emphasized that these programs focused on opportunities and conditions for safe sports participation rather than merely emphasizing restrictions. Finally, some participants expressed that advice and recommendations for sports participation are strongly dependent on the respective treatment center.

### 3.5 Societal level

This category was not included in Matlary et al.'s (32) original scheme, but it emerged repeatedly throughout the data material as in previous research on sports participation and visually impaired students (36). It referred to situations in which participants described themselves as ‘different’, including any consequences this may have. In doing so, participants compared themselves to a presumed societal ‘normality.’

On the one hand, depictions related to *perceived derivations from ‘normality’*. One participant stated about himself: ‘*And since I know myself that when I inject (name of medication), I am like a normal person for that period of time, I can actually do that [play soccer].*’ (Ben, FG 02). In this statement, Ben implicitly indicated that his perceived ‘normality’ occurred only through medication and is limited in time. Furthermore, several participants reported that they concealed their hemophilia from others because they wished to be treated ‘normally.’

The underlying *idea of normality* was primarily associated with the absence of hemophilia. Albeit participants also critically reflected on this notion. Steve expressed this perspective as follows: ‘*There isn't just the normal way, the healthy way, where you can do whatever you want. There are also other people, and they are normal too, but just differently normal, if you understand what I mean*.’ (Steve, FG 01).

This level can be understood as having a cross-sectional effect on the other levels, as societal narratives of normality shape experiences across diverse contexts. For this reason, recognizing and making this level visible appeared important in the analysis.

## 4 Discussion

This study examined the barriers and facilitators perceived and socially shared by yyalwh in relation to PA and sports participation. The results highlight that (non-)participation in sports is embedded across multiple, interrelated and sometimes even ambivalent levels. This finding is consistent with previous research on disability or chronic conditions and sports participation (35, 49). The following section discusses the results in relation to the levels and intertwined barriers and facilitators, with a particular focus on those levels that were most prominent in the data. Notably, the social level emerged as the most salient and is therefore discussed in greater depth below.

The data shows that many of the factors identified are positioned on a continuum. If, for example, the factor of access to the health care system is deficient or absent, this creates a barrier to daily life and thus also to participation in sports. When it is present and accessible, however, it can act as a facilitator. Between these two poles, different degrees and nuances become visible. In many situations sketched out by the participants, not only difficult circumstances were described, but also wishes and suggestions for improvement were frequently articulated. For example, several participants stated that their individual participation in sports should be taken into account when planning their treatment and that they would appreciate greater involvement in decision-making processes. In this regard, the desire for shared decision-making between patients and medical professionals became particularly evident (50). Similar results were also reported in the qualitative study by Cotino et al. (11).

When examining the data regarding the thematic focus of the situations described, it becomes clear that many of these situations were primarily situated at the social level. Both, qualitative studies on hemophilia with plwh and their parents (18) as well as qualitative research on other chronic conditions, such as asthma (51), likewise emphasize the considerable importance of social factors. Therefore, the following section explores in greater depth how the social level shapes barriers and facilitators to PA and sports and discusses the results in light of further literature.

In the focus groups, participants repeatedly described parents and treating physicians as gatekeepers who had the authority to decide whether to allow, maintain, or restrict participation in sports. While participants who were minors and of school-age described parental care as particularly influential for their everyday decisions, those who already reached legal age described themselves as primarily responsible for their treatment and decisions related to physical activity, usually in consultation with their treating doctors. This observation partly aligns with findings of Potì et al. (15), who used qualitative interviews to map central aspects of living with hemophilia across age groups and treatment regimens. Their analysis revealed age-related differences: participants aged 11–15 years tended to place their bodies and experienced physical sufferings at the center of their narratives, those aged 16–19 years primarily brought up questions about the impact of hemophilia on their attitudes and close social relationships, and those aged 20–25 years emphasized questions of treatment self-management. These findings indicate that responsibility and decision-making processes shift with increasing age and autonomy. However, during childhood and adolescence, when parents still play a major role in these decisions, it is important to acknowledge the complex emotions they may experience regarding their child's diagnosis. For example, Onel et al. (52) found that parents reported feelings of guilt and responsibility, which in turn influenced everyday family life and family dynamics. Furthermore, Williams and Chapman (18) showed that parents’ increased ‘awareness of difference’ (p. 203) often results in particularly strong protective practices. The findings presented suggest that such (over-)protective behaviors may simultaneously limit opportunities for self-determined sports participation.

In addition to parents and doctors, participants also mentioned a wider range of social actors who influence their participation in sports, including other relatives, teachers, coaches, friends, and classmates. Primary and wider family as well as experts (psychologists, healthcare providers, teachers) and friends have also been identified as relevant social influences in previous research (53). Unlike medical professionals, coaches and PE teachers were partly perceived by participants as having limited knowledge about hemophilia. The invisibility of hemophilia therefore emerged as a key factor, as hemophilia is not immediately apparent to others. Participants reported that, often and in many cases at the request of their parents, they themselves had to explain their chronic condition to their PE teachers. The broader need for disclosure and explanation towards external social actors has also been identified as an important psychosocial issue in previous research (15, 54). Peer interactions were also considered important by the participants. Support from peers was described as highly valuable, a finding also reported by Sterling et al. (55), whereas stigmatizing situations, particularly those demanding justification for non-participation, were experienced as burdensome. This points out that invisibility and rarity of hemophilia do not necessarily reduce stigma but often produce additional interactional work (10).

Furthermore, participants repeatedly discussed their described situations in terms of ‘normality’ and ‘deviation’, mirroring previous qualitative research on hemophilia (18). Although these aspects were not asked for by the moderators of the focus groups, participants themselves made statements referring to (deviations from) ‘normality’. Their discussions were closely linked to critical reflections on normative, able-bodied performance imperatives and assessment systems effective in school. Ableist assumptions were occasionally reproduced among participants themselves, a pattern comparable to findings in other research contexts related to disability, specifically visual impairment, and sports (56). This was expressed through comparisons between themselves and an external ‘healthy group.’ In some cases, the self-description as ‘hemophilic’ was accompanied by reduced expectations of their own abilities. This reflects forms of internalized ableism (57, 58). These self-descriptions and perceptions are highly relevant regarding adequate psychosocial support. Regarding possible psycho-educational actions, Potì et al. (15) also note that particularly young children with hemophilia should develop a ‘perception of being “lived-in bodies,” not just “bodies under care” ‘ (p. 140). To achieve this, effective societal normative assumptions and ability-related narratives must be recognized and critically reflected upon by all actors involved. Concerning this, factors at the societal level operate across the other levels. Therefore, raising awareness and educating key actors, such as parents, doctors, and teachers is essential (59, 60).

Examining existing ableism narratives in greater depth could therefore be a key objective of a subsequent research project. Furthermore, future research itself should be designed in an inclusive and ableism-critical manner (61). Participatory research approaches primarily meet this demand, allowing yyalwh themselves to become active members of the research team and to play a decisive role in shaping interpretations.

## 5 Limitations

This study has several limitations. First, the sample represents a group with a strong affinity to sports. Almost all participants engage in sports regularly, and some are members of organized sports structures, for example as members of sports clubs. Consequently, this sample does not include the perspectives of individuals living with hemophilia who may have a rather negative attitude towards PA. Furthermore, the analysis was conducted at an overarching level without differentiating between hemophilia types and severities or between age groups. If analyzed separately, there might be specific patterns of results along those categories mentioned. However, given the novel focus on examining sports participation from the perspective of yyalwh themselves, the decision was made to first conduct a general, overarching analysis rather than introducing an artificial separation into subgroups. Nevertheless, a more differentiated analysis could be undertaken in future work. Additionally, it may be criticized that no further demographic data were collected, which limits the possibility of conducting a more differentiated analysis across specific thematic dimensions. However, since this was a qualitative study, the aim was not to generate statistically generalizable findings but to explore participants’ experiences in the context of participation in PA and sports. Future quantitative studies could examine the extent to which the barriers and facilitators identified in this study are reflected in larger and more diverse populations of plwh. Finally, it must be mentioned that this study does not include participatory research elements, particularly regarding data analysis. Although the planning and conception of the project proactively involved participants, the research team nevertheless consisted of members of the academic community. It remains a goal to summarize the study's findings and publish them in the patient organization's journals so that the empirically derived results may reach the community itself.

## 6 Conclusion

Considering Article 8 (awareness-raising) and Article 30 of the UN-CRPD, which requires signatory countries to ensure equal access to cultural life, recreation, leisure and sport, a critical look at current conditions for participation in PA and sports for yyalwh is a necessary first step.

In the study, the participants emphasized that medical treatment is constantly evolving and expanding the possibilities for participation in PA and sports. However, access to and participation in sports do not only depend on medical advances but are significantly shaped by social factors. In this sense, support from immediate social actors, particularly parents, doctors and teachers, proves to be a major enabling factor. At the same time, participation can also be constrained by social dynamics. Explicit restrictions imposed by social actors, operating as gatekeepers, as well as the recurring demand for self-disclosure and explanations concerning hemophilia, were described by the participants as hindering factors.

Especially noteworthy is furthermore the societal level, which exerts an influence on all the other levels. Participants described situations in which they themselves, as well as actors in their (immediate) social environment, negotiated hemophilia in relation to a perceived deviation from (body-related) ‘normality’. Ableist narratives became apparent both in the participants’ self-description and their descriptions of lived experiences. From an ableism-critical perspective, it is therefore crucial to examine these narratives in greater depth. Doing so can contribute to raising awareness among diverse stakeholders and to fostering a critical controversy about existing societal norms and assumptions that shape perceptions of (deviant marked) bodies.

## Data Availability

The datasets presented in this article are not readily available as it consists of qualitative interview transcripts that contain personally identifiable information about the participants. Due to confidentiality agreements and ethical considerations, the complete dataset cannot be shared in order to protect the participants’ privacy. Please direct any inquiries regarding access to the datasets to TN, tabea.nauschuetz@uni-marburg.de.
